# Exogenous Hormone Factors in Relation to the Risk of Malignant Melanoma in Women: A Systematic Review and Meta-Analysis

**DOI:** 10.3390/cancers14133192

**Published:** 2022-06-29

**Authors:** Manuela Chiavarini, Giulia Naldini, Irene Giacchetta, Roberto Fabiani

**Affiliations:** 1 Department of Experimental Medicine, Section of Public Heath, University of Perugia, 06129 Perugia, Italy; manuela.chiavarini@unipg.it; 2 School of Specialization in Hygiene and Preventive Medicine, University of Perugia, 06129 Perugia, Italy; naldini.giulia@gmail.com; 3 Department of Chemistry, Biology and Biotechnology, University of Perugia, 06129 Perugia, Italy; roberto.fabiani@unipg.it

**Keywords:** oral contraceptive, hormone replace therapy, melanoma, meta-analysis

## Abstract

**Simple Summary:**

Many epidemiological studies have examined the relationship between cutaneous malignant melanoma (CMM) and both endogenous oestrogen exposure (e.g., age at menarche and parity) and exogenous hormone use (e.g., oral contraceptives (OCs) and menopausal hormone therapy (MHT)). Though a previous meta-analysis investigating the relationship between characteristics of female endocrine status and CMM risk found no significant association, the potential role of THERAPY AS oral contraceptive (OC) and hormonal replacement therapy (MHT) use still remains controversial. Since then, several studies have been published about the therapy with contrasting results, while CMM incidence continues to increase with a significant gender divergence. The therapy of OC and MHT may play a role in CMM and the removal of this could be useful as emerging therapeutics in melanoma. Therefore, we conducted this systematic review and meta-analysis to summarize the evidence and derive a more accurate estimation of exogenous hormone factors in women and CMM.

**Abstract:**

The influence of exogenous female hormones on the risk of developing malignant melanoma in women remains controversial. The aim of our review and meta-analysis is to summarize the evidence and derive a more accurate estimation of the association between oral contraceptives (OCs) or menopausal hormone therapy (MHT) and the risk of developing malignant melanoma in women. PubMed, Web of Science, and Scopus database were searched for studies published up until October 2021. The PRISMA statement and MOOSE guidelines were followed. Studies were pooled using a random effects model. Heterogeneity was explored with the chi-square-based Cochran’s Q statistic and the I^2^ statistic. Publication bias was assessed with Begg’s test and Egger’s test. Forty-six studies met the eligibility criteria. The pooled analysis (26 studies) on OC use and the risk of developing cutaneous malignant melanoma (CMM) showed no significant association, but demonstrated significant association for cohort studies (OR 1.08, 95% CI 1.01–1.16; I^2^ = 0.00%, *p* = 0.544). The pooled analysis (16 studies) showed a significantly increased risk of CMM in association with MHT (OR 1.15, 95% CI 1.08–1.23; I^2^ = 25.32%, *p* = 0.169). Stratifying the results by study design showed that a significant increased risk of CMM was associated with MHT in the cohort studies (OR 1.12; 95% CI 1.04–1.19; I^2^ = 0%, *p* = 0.467). No significant publication bias could be detected. Further studies are needed to investigate the potential association with formulation, duration of use, and dosage of use, and to better understand the role of possible confounders.

## 1. Introduction

Cutaneous malignant melanoma (CMM) is the sixth most common cancer in women worldwide [1], with an estimated 137,000 (129,800–144,600) new cases in 2018 [2] and represents the 16th cause of cancer death [1].

A considerable decline in mortality rates was observed in the period 2013–2017 (6.3% per year), while incidence rates increased by 1.9% (1.5–2.2) per year [1].

Ultraviolet radiation exposure [3,4]; naevi (common and atypical) count [5]; freckle density; phenotypic characteristics (skin type I, skin color, eye color, and hair color) [6]; a family history of melanoma [6,7]; and familiar susceptibility due to low-, medium-, or high-penetrance genes [8] are well-established risk factors for CMM. Intriguingly, several pieces of epidemiological data have noted a significant gender divergence in CMM incidence [1,2,9]. Particularly, the incidence of CMM is higher in adolescent and young adult females [10]. Compared to males, the probability of developing CMM increases in women under the age of 50, but is lower at an older age [9]. Moreover, a gender difference in CMM survival has been noted for the early stage of the disease, though results are controversial in more advanced stages [11,12].

Following the observation of these sex differences, many epidemiological studies have examined the relationship between CMM and both endogenous estrogen exposure (e.g., age at menarche and parity) and exogenous hormone use (e.g., oral contraceptives [OCs] and menopausal hormone therapy [MHT]) [13,14,15,16,17,18,19,20,21,22,23,24,25,26,27,28,29,30,31,32,33,34,35,36,37,38,39,40,41,42,43,44,45,46,47,48,49,50,51,52,53,54,55,56]. Though a previous meta-analysis [57] investigating the relationship between characteristics of female endocrine status and CMM risk found no significant association, the potential role of oral contraceptive (OC) and hormonal replacement therapy (MHT) use remains controversial.

Since then, several studies have been published with contrasting results. Therefore, we conducted this systematic review and meta-analysis to summarize the evidence and derive a more accurate estimation of malignant melanoma risk and exogenous hormone factors in women.

## 2. Materials and Methods

This systematic review and meta-analysis were conducted and reported according to the meta-analysis of observational studies in epidemiology (MOOSE) guidelines [58] and the preferred reporting items for systematic reviews and meta-analyses (PRISMA) statement [59].

### 2.1. Search Strategy and Data Source

We carried out a comprehensive literature search, without restrictions, up until 1 October 2021 through PubMed (http://www.ncbi.nlm.nih.gov/pubmed/, accessed on 28 April 2022), Web of Science (http://apps.webofknowledge.com, accessed on 28 April 2022), and Scopus (https://www.scopus.com/, accessed on 28 April 2022), databases to identify all the original articles investigating the association between exogenous hormone use and malignant melanoma risk in women. The following search medical subject headings (MeSH) and key words were used: (“oral contraceptive” OR “exogenous hormones” OR “hormonal therapy” OR “hormone therapy”) AND (melanoma OR “skin cancer”). In addition, the reference lists of included articles and recent relevant reviews were manually examined to identify additional relevant publications.

### 2.2. Eligibility Criteria

Publications were eligible if they: (i) evaluated the relationship between exogenous hormone use and malignant melanoma in women; (ii) used a case–control, prospective, or cross-sectional study design; (iii) presented risk estimates (odds ratio, OR; relative risk, RR; or hazard ratio, HR) with 95% confidence intervals (CIs). In the presence of several publications from the same study, the publication with the biggest sample was selected. For each potentially included study, two investigators independently conducted the selection, data abstraction, and quality assessment. Disagreements were resolved by discussion or in consultation with a third author. Although it is useful to have background information, reviews and meta-analyses were excluded. No studies were excluded based on weakness of design or data quality.

### 2.3. Data Extraction and Quality Assessment

From the included studies, we extracted the following information: the first author’s last name, the year of publication, country, the study design, the sample size (when possible, the number of cases and controls and incident cases, as well cohort size), population characteristics (age, ethnicity), the duration of follow-up for cohort studies, tumor characteristics (CMM; superficial spreading melanoma, SSM; nodular melanoma, NM; and uveal/intraocular melanoma), the identification of cases, exposure assessment, OCs exposure (the duration of use, the time since the most recent OC use, the time since the first OC use, the status of OCs, and the age at first use), MHT exposure (the duration of use, the status of MHT use, regimen, the type of MHT, and the route of administration), risk estimates with 95% CIs for the different categories of exogenous hormone use, a *p*-value for trend, and adjustment of confounding factors. When multiple estimates were reported in the article, those adjusted for the most confounding factors were pulled out. The Newcastle–Ottawa Scale (NOS) [60] was used for the quality evaluation of the enrolled studies. NOS adopted a star system, with a total score ranging from 0 to 9. A total score of ≥7 indicated a high-quality study. Two investigators individually performed the quality evaluation of each selected study and disagreements were settled by a joint reevaluation of the original article with a third author.

### 2.4. Statistical Analysis

We evaluated the association between exogenous hormone use (OCs and MHT) and malignant melanoma’s risk in women using the statistical program ProMeta version 3.0 (IDo Statistics-Internovi, Cesena, Italy). For the overall estimation, the relative risk and hazard ratio were taken as an approximation to the OR, and the meta-analysis was performed as if all types of ratio were ORs. The combined risk estimate was calculated using a random effect model.

The chi-square-based Cochran’s Q statistic and the I2 statistic were used to evaluate heterogeneity in results across studies [61]. The I2 statistic yields results ranged from 0% to 100% (I2 = 0–25%, no heterogeneity; I2 = 25–50%, moderate heterogeneity; I2 = 50–75%, large heterogeneity; and I2 = 75–100%, extreme heterogeneity) [62]. Results of the meta-analysis may be biased if the probability of publication is dependent on the study results. We used the method by Begg and Mazumdar [63] and the method by Egger et al. [64] to detect publication bias. Both methods were tested for funnel plot asymmetry—the former was based on the rank correlation between the effect estimates and their sampling variances, and the latter was based on a linear regression of a standard normal deviate on its precision. If a potential bias was detected, we further conducted a sensitivity analysis to assess the robustness of combined effect estimates, and the possible influence of the bias, and to have the bias corrected. We also conducted a sensitivity analysis to investigate the influence of a single study on the overall risk estimate, by omitting one study in each turn. We considered the funnel plot to be asymmetrical, if the intercept of Egger’s regression line deviated from zero, with a *p*-value < 0.05.

## 3. Results

### 3.1. Study Selection

**Figure 1:**
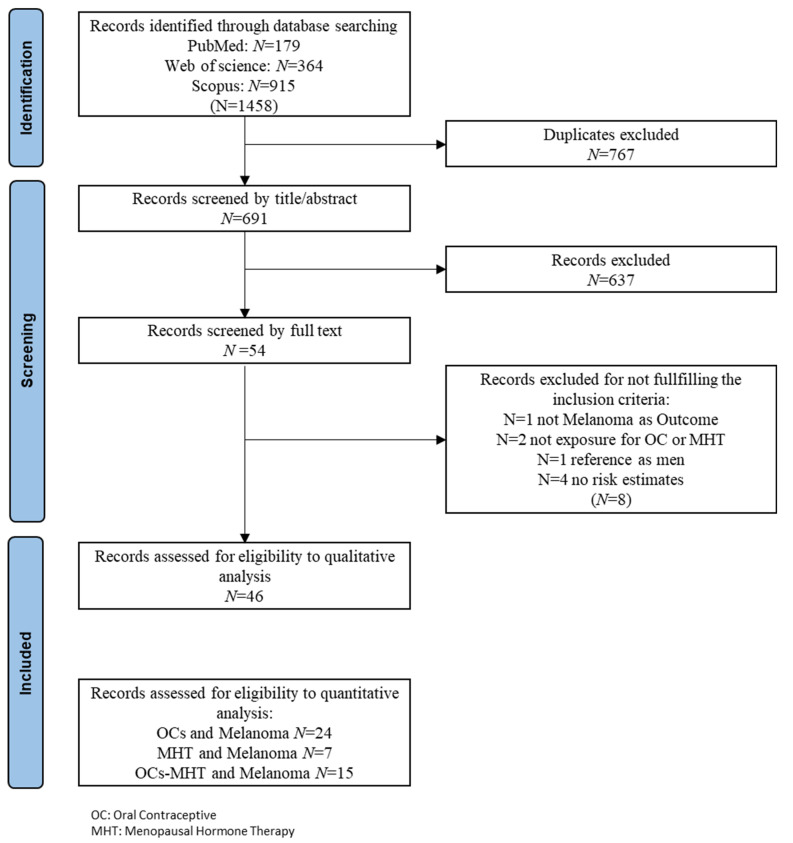
PRISMA flow chart of included studies.

The study selection process is shown in Figure 1. The primary literature research through PubMed (*n* = 179), Web of Science (*n* = 364), and Scopus (*n* = 915) databases returned a total of 1458 records. Duplicates (*n* = 767) were removed. Based on the title and abstract revision, we identified 54 eligible records on exogenous hormone use and malignant melanoma in women. Hand searching of reference lists of both already selected articles and recent relevant reviews led to the identification of no additional item. Of the 54 records subjected to full-text revision, 8 were further excluded because they failed to meet the inclusion criteria (1 did not report malignant melanoma as an outcome, 2 studies did not report exposure for OCs or MHT, 1 used men as a reference group, and 5 reported no risk estimates).

Therefore, at the end of the selection process, 46 studies were eligible for final inclusion in the systematic review and meta-analysis. Of these, 15 studies reported risk estimation of both OCs and MHT for malignant melanoma in women. Thirty-nine records investigated the relationship between OCs and malignant melanoma in women and twenty-two records investigated the relationship between MHT and malignant melanoma in women.

### 3.2. Meta-Analysis on the Risk of Developing Malignant Melanoma and OC Use

#### 3.2.1. Study Characteristics and Quality Assessment

The detailed characteristics of the studies on the association between OCs and malignant melanoma are shown in Table A1. Among the 39 selected studies [13,15,16,17,20,22,23,24,25,26,27,28,29,30,31,32,33,35,36,37,38,39,40,41,42,43,44,45,46,47,49,50,51,52,53,54,55,56,65], 25 are case–control studies [13,15,16,17,27,28,29,32,33,35,36,37,38,39,41,42,43,44,45,46,49,53,54,55,56] and 14 are cohort studies [20,22,23,24,25,26,30,31,40,47,50,51,52,65].

The evaluated outcomes in this meta-analysis were CMM, superficial spreading melanoma (SSM), nodular melanoma (NM), and uveal/intraocular melanoma. Thirty-five studies [13,15,17,22,23,24,25,26,27,28,29,30,31,33,35,36,38,39,40,41,42,43,44,45,46,47,49,50,51,52,53,54,55,56,65] investigated the risk of CMM associated with OCs, 9 studies [22,27,28,35,36,38,39,46,55] investigated the risk of SSM associated with OCs, and 7 studies [22,27,28,35,38,39,46] investigated the risk of NM associated with OCs. Three studies [16,32,37] analyzed the risk of uveal/intraocular melanoma associated with OCs and one study (20) analyzed the risk of melanoma associated with OCs. Regarding the association between CMM and OCs, 26 studies [13,15,17,22,23,27,28,29,30,31,33,35,36,38,39,40,42,44,45,47,50,51,52,54,55,56] included cases of in situ and invasive melanomas, whereas 7 studies [24,25,26,41,43,49] selected invasive melanomas only. The study by Palmer et al. [46] referred to severe invasive cutaneous melanoma. Thirty-four studies [13,15,17,22,23,24,25,26,27,28,29,30,31,33,35,36,38,39,40,41,42,43,44,45,46,47,49,50,51,52,53,54,55,56] reported risk estimates for SSM, three studies [16,32,37] for uveal/intraocular melanoma, and one study [20] for melanoma. Sixteen studies [13,20,22,23,24,31,35,36,38,45,47,53,54,55,56,65] assessed the outcome through record linkage to cancer registries, thirteen studies [17,26,27,28,29,32,33,39,41,42,43,44,49] assessed the outcome through histology and/or pathology confirmation, two studies [16,25] collected outcome information from general practitioners (GP) records, five studies [37,46,50,51,52] collected outcome information from the hospital records, one study [15] collected outcome information from either a pathology report or hospital discharge notes, and one study [40] did not specify the source of information. The study by Hannaford et al. [30] collected outcome information from GP records in one cohort and from hospital discharge record in the other one. Twenty-three studies [17,27,28,29,32,33,35,36,37,38,39,42,43,44,45,46,49,50,51,52,55,65] assessed OCs exposure through an interview, eight studies [15,16,22,23,24,25,26,53] through the administration of a questionnaire, and two studies [13,30] through either a questionnaire or an interview, while two studies [13,31] collected information from GP or medical records and one study [47] from pharmacy records, and the study by Koomen et al. [41] extracted data from the national registry. No information on exposure assessment was available in the study by Kay et al. [40]. Nine studies [22,25,29,31,38,42,46,51,52] reported risk estimates related to the time since the most recent OC use, seven studies [22,23,25,26,42,46,49] to the age at the first use, five studies [22,25,26,46,50] to the status of OC use, and four studies [25,38,46,65] to the time since the first use. Twenty studies [13,15,17,25,26,27,28,29,30,31,32,33,36,37,40,45,46,50,51,52] reported risk estimates as RR, thirteen studies [16,35,38,39,41,42,43,44,49,53,54,55,56] as OR, and five studies [20,22,23,24,65] as HR, whereas one study [47] reported SIR. One study [24] referred to never-users for all cases that have never used OCs or that have used OCs for less than a year.

The study-specific quality scores of selected studies are shown in the last column on the right of Table A1. The quality scores ranged from 0 to 8 (median: 6; mean: 6.1). The median values of cohort studies and case–control studies were seven and six, respectively. Among cohort studies, ten records [20,22,23,24,30,31,47,51,52,65] had a high score, three [25,26,30] had a medium score, and one study [40] had a low score. Eight case–control studies [35,37,41,43,44,45,49,54] had a high score, sixteen case–control studies [13,15,16,27,28,29,32,33,36,38,39,42,46,53,55,56] had a medium score, and one [17] had a low score.

#### 3.2.2. Meta-Analysis

**Figure 2:**
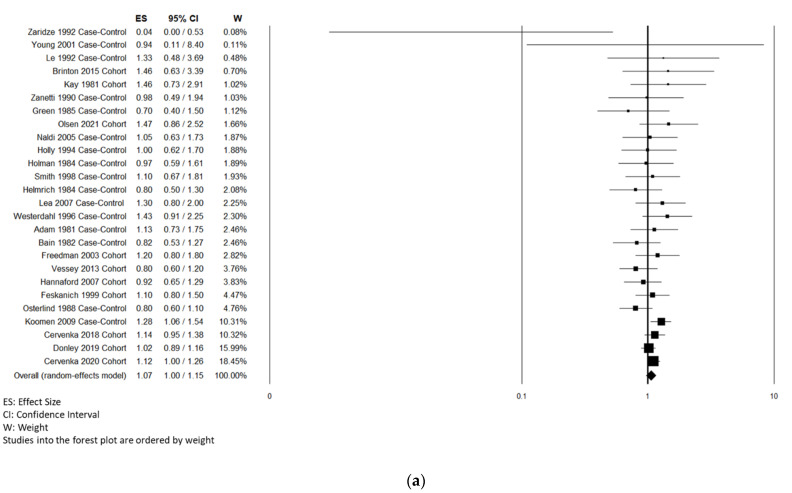

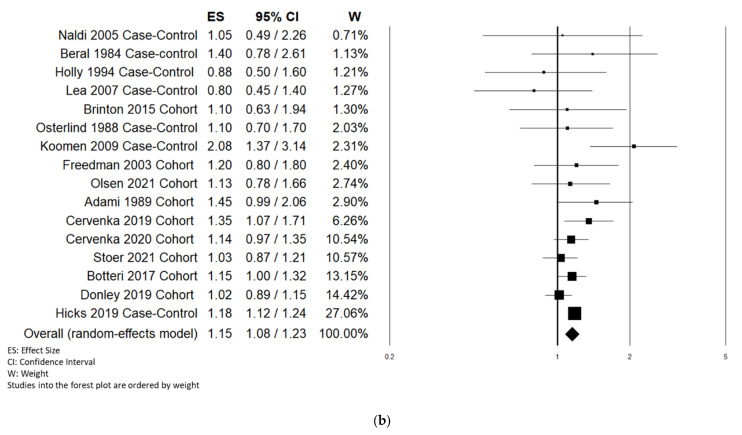
Forest plot of OC (**a**) and MHT (**b**) use and risk of CMM.

Twenty-six studies [13,15,20,22,23,24,25,26,29,31,33,37,39,40,41,42,43,44,45,49,52,53,54,55,56,65] included in the systematic review were used for the overall risk estimation of CMM (Table 1, Figure 2a). One study [47] was excluded as reporting SIR and no risk estimates. In the overall analysis, OC use did not significantly affect the risk of developing CMM. Stratifying the results by study design, the time since the most recent OC use, and status of use showed no significant association between the risk of developing CMM and OC use. The stratification by study design showed a significant association for cohort studies; the stratification by age at the first use showed a significant association for an age greater than 20 years old. Stratifying the analysis by melanoma morphology showed that OC use did not significantly affect the risk of developing SSM or NM. In the overall analysis, the risk of developing uveal/intraocular melanoma showed no significant association with OC use.

#### 3.2.3. Sensitivity Analyses

Sensitivity analyses suggested that the estimates were slightly modified by any single study. In particular, a small change was found in the risk estimates after removing the study by Koomen et al. [41] (OR: 1.05; 95% CI: 0.99, 1.13; *p* = 0.239). However, removing the study by Østerlind et al. [45] resulted in a small increment of melanoma risk, which became statistically significant (OR: 1.09; 95% CI: 1.02, 1.16; *p* = 0.008).

#### 3.2.4. Publication Bias

**Figure A1:**
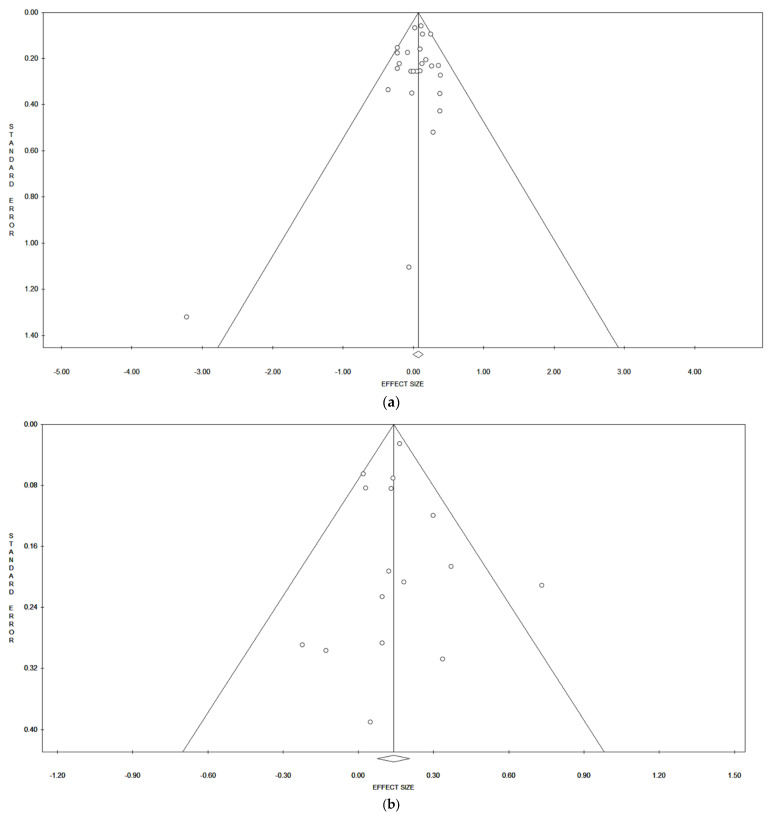
Funnel Plot of publication bias of OC (**a**) and MHT (**b**) use and risk of CMM.

No significant publication bias was detected with Egger’s or Begg’s tests (Table 1, Figure A1).

### 3.3. Meta-Analysis on the Risk of Malignant Melanoma and MHT Use

#### 3.3.1. Study Characteristics and Quality Assessment

The detailed characteristics of the studies on the association between MHT and malignant melanoma are shown in Table A2.

Among the 22 selected studies [14,16,17,18,19,20,21,23,24,26,32,34,35,36,37,41,43,44,45,48,65,66], 11 are case–control studies [16,17,32,34,35,36,37,41,43,44,45] and 11 are cohort studies [14,18,19,20,21,23,24,26,48,65,66].

The evaluated outcomes in this meta-analysis were CMM and uveal/intraocular melanoma. Eighteen studies [14,17,18,19,21,23,24,26,34,35,36,41,43,44,45,48,65,66] investigated the risk of CMM associated with MHT, three studies [16,32,37] investigated the risk of uveal/intraocular melanoma associated with MHT, and one study [20] investigated the risk of melanoma with MHT. Regarding the association between the risk of CMM and MHT, 10 studies [14,17,18,21,23,35,36,44,45,48] included cases of in situ and invasive melanomas, whereas 6 studies [19,24,26,34,41,43] selected invasive melanomas only. Three studies reported risk estimates for SSM [21,35,36], two studies [21,35] reported risk estimates for NM, and one study [21] reported risk estimates for LMM and ALM. Fourteen studies [14,18,19,20,21,23,24,34,35,36,45,48,65,66] assessed the outcome with record linkage to cancer registries, six studies [17,26,37,41,43,44] assessed the outcome with histology and/or pathology confirmation, one study [16] collected outcome information from general practitioners’ records, and one study [32] collected outcome information from the ocular oncology unit. Ten studies [16,17,32,35,36,37,43,45,65,66] assessed MHT exposure with an interview, six studies [14,21,23,24,26,44] with the administration of a questionnaire, and one study [20] with a questionnaire and medical records, whereas five studies [18,34,41,48] used a national registry or a database of drug prescriptions, and one study [19] used the medical reimbursement register of the national social insurance. Eleven studies [19,21,23,24,32,35,36,37,41,45,65] reported risk estimates for the duration of MHT use, four studies [18,21,23,66] for the status of MHT use, two studies [18,23] for regimen therapy, seven studies [14,18,21,23,45,48,66] for the type of MHT, and three studies [21,23,34] for the route of administration. Nine studies [14,17,18,26,32,36,37,45] reported risk estimates as RR, six studies [16,34,35,41,43,44] as OR, six studies [20,21,23,24,65,66] as HR, and two studies [19,48] reported SIR. One study [34] used as reference a category named non-users, which included patients who did not use MHT (excluding intravaginal estrogens) in the five years prior to diagnosis and one year after diagnosis.

Table A2 shows study-specific quality scores of the selected studies. The quality scores ranged from 3 to 9 (median: 7; mean: 6.3). The median value for both cohort studies and case–control studies was seven. Among cohort studies, eight records [18,19,20,21,23,24,65,66] had a high score and three [14,26,48] had a medium score. Six case–control studies [34,35,41,43,44,45] had a high score, four case–control studies [16,32,36,37] had a medium score, and one [17] had a low score.

#### 3.3.2. Meta-Analysis

Sixteen studies [14,17,18,20,21,23,24,26,34,35,41,43,44,45,65,66] included in the systematic review were used for the overall risk estimation of CMM (Table 2, Figure 2b).

Two studies [19,48] were excluded as reporting SIR and no risk estimates. We found that the risk of developing CMM was significantly higher in ever-users of MHT (OR 1.15, 95% CI 1.08–1.23). Stratifying the results by study design showed a significantly increased risk of CMM in cohort studies only (OR 1.12, 95% CI 1.04–1.19). Current MHT users had a significant higher risk (+19%) of CMM. Stratifying the analysis for the route of administration showed a significantly increased risk of CMM for both oral administration (OR 1.19, 95% CI 1.11–1.27) and, more noticeably, transdermal–cutaneous administration (OR 1.36, 95% CI 1.19–1.54). Stratifying the results by the type of MHT showed a significant positive association with the risk of developing CMM of ET only (OR 1.34, 95% CI 1.18–1.52). No significant association with the duration of MHT use was found.

Three studies [18,21,23] included in the systematic review were selected for the overall risk estimation of uveal/intraocular malignant melanoma. MHT use showed no significant association with the risk of developing uveal/intraocular malignant melanoma.

#### 3.3.3. Sensitivity Analyses

Sensitivity analyses investigating the influence of a single study on the CMM risk estimates suggested that these were not substantially modified by any single study. Indeed, the CMM risk estimates ranged from 1.14 (95% CI 1.07–1.24, *p* = 0.0001), omitting the study of Cervenka et al. [21], to 1.17 (95% CI 1.10–1.25, *p* < 0.0001), omitting the study of Donley et al. [24]

#### 3.3.4. Publication Bias

No significant publication bias was detected with Egger’s or Beggs method (Table 2, Figure A1).

## 4. Discussion

The incidence of cutaneous melanoma continues to increase globally [67], presenting a challenge in identifying unestablished risk factors. Melanoma is classically considered a non-hormone-related cancer; nevertheless, cutaneous melanoma has been widely investigated as a steroid hormone-sensitive cancer (particularly estrogens) [68]. Indeed, female hormones can contribute to modulate cellular proliferation and cell cycle progression through receptor-mediated transcriptional mechanisms [69]; moreover, previous studies reported the expression of progesterone and estrogen receptors in melanoma in various degrees [68,70]. Evidence suggests that estrogens may contribute to the gender differences in the immune pathways [71] and response [72,73], even though the role of sex hormones in the immunologic escape of cancer remains unclear [74,75]. Steroid hormones such as estrogen act through their cognate receptors, i.e., estrogen receptor alfa (ERα) and estrogen receptor beta (ERβ) [76]. ERs belong to the nuclear receptor superfamily, which act as transcription factors. Estrogen binding to the nuclear receptors is responsible for a nuclear translocation, with the consequent activation of genomic pathways and the transcription of multiple target genes. ERα promotes DNA transcription, while ERβ inhibits it; ERα plays a role in tumorigenesis by stimulating cell proliferation, while ERβ seems to have a significant antitumor activity [77,78]. When ERs are linked to the G protein of cellular membrane molecules, i.e., the G-protein-coupled estrogen receptor (GPER), ERs act as membrane receptors via a “non-genomic pathway”. GPER are responsible for changes in the cytosolic signaling, leading to increased activity of the RAS/BRAF/MEK axis. The GPER acts via intracellular cAMP-protein kinase (PK) and cAMP-response element-binding protein (CREB) phosphorylation. GPERs are involved in the development and progression of different cancer types. In skin, GPERs regulate melanin production and are expressed in melanoma cells. They promote melanogenesis and regulate melanocyte growth, differentiation, and function [78,79]. In conclusion, the correlation between endogenous female hormones and cutaneous melanoma has been extensively studied [49,80,81,82,83], while the potential link between exogenous female hormones, either OCs or MHT, and CMM development has only been recently investigated. This underlines the importance of investigating the influence of different types of exogenous hormones and the risk of developing CMM.

Our systematic review and meta-analysis summarized the evidence and investigated the effect of exogenous hormones on the risk of developing melanoma in women. Our analysis showed no significant association between OC use and the risk of developing CMM, and our findings agree with threeprevious meta-analyses [57,84,85].

The use of the exogenous hormone, in accordance with our meta-analysis, does not affect the risk of developing SSM or NM, even if it should be considered that this result could be influenced by a small number of studies included for SSM and NM.

Our results for OC use and the risk of developing CMM are in accordance with the recent meta-analysis of Sun et al., 2020, which is based on twenty-seven studies [85]; however, it included even letters to the editor and excluded two case–control studies [29,40] and a large cohort study [20], which was considered within our review instead.

In contrast to the previous meta-analysis by Gandini et al. [57], our meta-analysis showed a significant association between MHT and an increased risk of CMM. It is noteworthy that our meta-analysis included two multicentric studies and five cohort studies, which were excluded by Gandini et al. [57]. Our results on MHT use and the risk of developing CMM are in accordance with the two most up-to-date meta-analyses [85,86]; in fact, both suggested that the use of MHT is related with an increased risk of developing melanoma in women. In particular, our results are in accordance with Sun’s (2020) and Tang’s (2020) results in relation to hormone type (estrogen), and with Sun’s (2020) results in relation to study type (cohort). However, these two meta-analyses [85,86], as already described in two different letter to the editor [87,88], do not include three large cohort studies [19,20,48] considered within our meta-analysis. Stratifying the analysis by study design demonstrated that the increased risk of CMM in association with MHT was confirmed among prospective cohort studies, which are less prone to bias compared with retrospective studies. The type of MHT, the route of administration, and the current status of use seemed to play a role in increasing the risk of developing CMM. Our findings referring to the type of MHT suggest that exogenous estrogen presents a risk factor for CMM, while the formulations of MHT containing estrogen and progestin showed no significant association with the risk of developing CMM.

### Limitations

We are aware that our analysis has several limitations and that caution is needed in interpreting our findings. Firstly, we could not investigate the OC formulations, which differed considerably during the years of publication of the included studies. The meta- analysis on OC use and the risk of developing CMM included 12 studies published in the 1980s, 12 studies published in the 1990s, 8 studies published in the 2000s, and 6 studies published in the 2010s. Secondly, we found substantial heterogeneity among the studies, despite the availability of many relevant papers. Thirdly, the observed association between MHT and CMM risk could be partially due to unmeasured or residual confounding, although the majority of the selected studies reported risk estimates adjusted for major potential confounders (e.g., age, body mass index, smoking, pigmentary traits, and parity). Furthermore, the stratified analyses on the type of MHT, the route of administration, and the status of use were performed on a small number of risk estimates. We did not stratify the results by age, which represents a major confounding factor for the association between hormonal/reproductive factors and cancer risk. Lastly, all the included studies reported a risk estimation for CMM in Western populations. Ethnic differences are not only potentially related to pigmentary traits, but also to differences in the use of OCs or MHT, contributing to risk effects associated with CMM.

More studies are needed to further investigate the potential role of MHT or OC formulation, the duration of use, the dosage of use, the age at first and last use, as well as the cancer receptor subtype [80,86].

## 5. Conclusions

In summary, our meta-analysis showed an increased risk of CMM in women receiving MHT, while no significant association between OC use and risk of developing CMM was found. The role of exogenous hormones in CMM tumorigenesis remains controversial. Further studies are needed to investigate the potential correlations of the dosage, duration of use, and formulation of OCs and MHT with risk of CMM, and to better understand the role of potential confounders, including age at first and last use and ethnicity.

## Figures and Tables

**Table 1:** Results of stratified analysis of malignant melanoma risk estimates for use of oral contraceptives (OCs). Reference category: no OC users.

	Sample Size	Combined Risk Estimate		Test of Heterogeneity			Publication Bias	
		Value (95% CI)	*p*	Q	I^2^%	*p*	P (Egger’s Test)	P (Begg’s Test)
CMM								
All (*n* = 26)	989,210	1.07 (1.00–1.15)	0.062	28.45	12.12	0.288	0.277	0.774
Study design								
Case–control (*n* = 16)	15,085	1.03 (0.89–1.19)	0.688	20.52	26.90	0.153	0.055	0.368
Cohort (*n* = 10)	974,125	1.08 (1.01–1.16)	0.032	7.90	0.00	0.544	0.572	0.421
Time since last OC use								
<4–5 years (*n* = 3)	200,907	0.91 (0.71–1.16)	0.434	2.76	0.00	0.431	0.544	1.000
5–9 years (*n* = 3)	200,907	0.90 (0.72–1.14)	0.397	0.55	0.00	0.907	0.376	0.174
>10 years (*n* = 3)	200,907	0.94 (0.58–1.51)	0.787	16.46	81.77	0.001	0.684	1.000
Status of use								
Past (*n* = 4)	334,135	1.15 (0.98–1.34)	0.086	0.13	0.00	0.988	0.768	1.000
Current (*n* = 4)	334,135	1.46 (0.95–2.25)	0.085	3.55	15.40	0.315	0.651	0.497
Age at first use								
≥20 years (*n* = 3)	389,059	1.16 (1.02–1.33)	0.028	0.41	0.00	0.817	0.652	0.602
SSM								
All (*n* = 5)	83,996	1.15 (0.83–1.59)	0.396	11.53	65.30	0.021	0.720	0.327
Study design								
Case–control (*n* = 4)	4631	1.20 (0.72–2.01)	0.489	11.31	73.48	0.010	0.823	0.497
NM								
All (*n* = 4)	83,763	0.86 (0.51–1.44)	0.569	5.16	41.89	0.160	0.975	1.000
Study design								
Case–control (*n* = 3)	4398	0.81 (0.42–1.57)	0.538	4.89	59.07	0.087	0.878	0.602
Uveal melanoma and intraocular melanoma								
All (*n* = 3)	2269	0.86 (0.64–1.15)	0.298	0.45	0.00	0.797	0.926	0.602

Abbreviations: CMM—cutaneous malignant melanoma; NM—nodular melanoma; OCs—oral contraceptives; SSM—superficial skin melanoma.

**Table 2:** Results of stratified analysis of malignant melanoma risk estimates for menopausal hormone therapy (MHT) use. Reference category: no MHT users.

	Sample Size	Combined Risk Estimate		Test of Heterogeneity			Publication Bias	
		Value (95% CI)	*p*	Q	I^2^%	*p*	*p* (Egger’s Test)	*p* (Begg’s Test)
CMM								
Ever-users (*n* = 16)	1,434,366	1.15 (1.08–1.23)	<0.001	20.09	25.32	0.169	0.972	0.719
Study design								
Case–control (*n* = 7)	182,909	1.20 (0.98–1.47)	0.077	10.44	42.52	0.107	0.969	0.881
Cohort (*n* = 9)	1,251,457	1.12 (1.04–1.19)	0.001	7.67	0.00	0.467	0.169	0.677
Duration of use								
<5 years (*n* = 7)	405,704	1.10 (0.92–1.31)	0.285	10.34	41.95	0.111	0.614	0.881
>5 years (*n* = 7)	399,472	1.06 (0.96–1.17)	0.267	0.78	0.00	0.993	0.422	0.652
Status of MHT use								
Current users (*n* = 4)	975,710	1.19 (1.09–1.30)	0.001	0.25	0.00	0.970	0.327	0.497
Past users (*n* = 4)	975,710	1.09 (0.89–1.33)	0.418	0.36	67.95	0.025	0.134	0.174
Route of administration								
Oral (*n* = 3)	384,140	1.19 (1.11–1.27)	<0.001	1.34	0.00	0.511	0.224	0.602
Transdermal–cutaneous (*n* = 3)	384,140	1.36 (1.19–1.54)	<0.001	0.12	0.00	0.941	0.112	0.117
Type of MHT								
ET (*n* = 6)	999,574	1.34 (1.18–1.52)	<0.001	3.45	0.00	0.632	0.280	0.851
EPT (*n* = 5)	976,330	1.12 (0.97–1.30)	0.119	6.16	35.11	0.187	0.457	0.624
Uveal melanoma and intraocular melanoma								
All (*n* = 3)	2269	1.32 (0.75–2.33)	0.328	7.57	73.59	0.023	0.654	0.602

Abbreviations: CMM—cutaneous malignant melanoma; ET—estrogen therapy; EPT—estrogen–progestin therapy; MHT—menopausal hormone therapy.

## Appendix A

**Table A1:** Main characteristics of studies included in the systematic review and meta-analysis on oral contraceptive (OC) use and malignant melanoma risk.

First Author Year Location	Study Design Name and Population Cases/Controls Follow-Up Incident Cases Age	Tumor Characteristics	Identification of Cases	Exposure Assessment	OCs Exposures Duration of Use Time Since Last OCsUse Time Since First OC Use Status of OC Use Age at First Use	OR/RR/HR/SIR (95% CI)	*p* for Trend	Matched or Adjusted Variables	NOS Score
Olsen et al. 2021 Australia	Cohort study Qskin Sun and Health Study 21068 Age: 40–69 y 392 (incidence 0.019) Follow-up: 5.4 y	Invasive CMM and all CMM (invasive and in situ)	Linkage with Queensland Cancer Registry	Survey Qskin Sun and Health	OC use No Yes Duration of OC use 0–6 months 7–60 months 61–120 121–240 >241 months	Ref 1.47 (0.86–2.52) Ref HR 1.12 (0.60–2.09) HR 1.54 (0.84–2.81) HR 1.43 (0.78–2.63) HR 2.12 (1.11–4.04)	0.01	Age, highest level of education achieved, body mass index, smoking status, and measure of health service use	8
Cervenka et al. 2020 10 European country	Cohort study EPIC 334,483 women Age: 51.1 ± 9.7 y 1,696 incident cases (in situ and invasive melanoma) Follow-up: 13.9 y	CMM: C44 (ICD-O-2)	Linkage with population cancer and pathology registries, health insurance and hospital discharge records, national and regional mortality registries, and active follow-up through contacts with participants and their next of kin	Country-specific questionnaire items	Never-users Ever-users Duration of use ≤5 years >5 years Age at first use ≤20 years 21–23 years 24–29 years ≥30 years	Ref. HR 1.12 (1.00–1.26) HR 1.11 (0.97–1.26) HR 1.20 (1.04–1.36) Ref. HR 1.12 (0.87–1.43) HR 1.20 (0.94–1.53) HR 1.24 (0.94–1.64)	0.01 0.19	Center, age at recruitment, education, age at menarche, length of menstrual cycles, number of full term pregnancies, menopausal status, height, body mass index, and tobacco use	8
Cervenka et al. 2018 France	Cohort E3N (Etude Epidémiologique auprès de femmes de l’Education Nationale) *n* = 79,365 Age: 45–60 y 539 incident cases (in situ and invasive melanoma *) Follow-up: 13.4 y	CMM	French National Cancer Institute	Questionnaire	Never-users Ever-users Duration of use <10 years ≥10 years Time since last OC use Quartile 1 Quartile 2 Quartile 3 Quartile 4 Status of OC use Never-users Past Current Unknown Age at first use Quartile 1 Quartile 2 Quartile 3 Quartile 4	Ref. HR 1.14 (0.95–1.38) HR 1.10 (0.87–1.38) HR 1.33 (1.00–1.75) Ref. HR 1.00 (0.73–1.37) HR 1.03 (0.63–1.69) HR 1.39 (0.68–2.82) Ref. HR 1.16 (0.94–1.41) HR 0.74 (0.30–1.84) HR 0.67 (0.16–2.77) Ref. HR 0.75 (0.50–1.11) HR 0.68 (0.46–1.02) HR 0.56 (0.36–0.86)	0.06 0.56 <0.01	Age and stratified according to year of birth, residential UV exposure at birth and at inclusion, pigmentary traits, and family history of skin cancer	8
		SM, NM, Lentigo maligna, ALM, Other			SSM Never-users Ever-users NM Never-users Ever-users Lentigo maligna Never-users Ever-users ALM Never-users Ever-users Other types Never-users Ever-users	Ref. HR 1.06 (0.84–1.34) Ref. HR 1.14 (0.34–3.80) Ref. HR 1.45 (0.78–2.69) Ref. HR 2.42 (0.85–6.90) Ref. HR 1.29 (0.82–2.03)		Residential UV exposure at birth and at inclusion, pigmentary traits, and family history of skin cancer	
Donley et al. 2019 USA	Cohort NIH-AARP *n* = 165,651 Caucasian women Age: 62.2 ± 5.3 y 1061 incident cases (invasive melanoma) Follow-up: 15.5 y	CMM: codes C44.0–C44.9 (ICD-O-3 Topography) and codes 8720–8780 (ICD-O-3 M)	Record linkage with state cancer registries	Questionnaire	Never or <1 year Ever Duration of use 1–4 years 5–9 years ≥10 years	Ref. HR 1.02 (0.89–1.16) HR 0.95 (0.80–1.13) HR 1.06 (0.87–1.28) HR 1.09 (0.88–1.34)	0.41	Age, ambient ultraviolet radiation quartile, education, body mass index, smoking status, marriage, family history of cancer, colonoscopy or sigmoidoscopy, and menopausal hormone therapy	7
Brinton et al. 2015 USA	Cohort *n* = 9892 women 70 incident cases Median follow-up 30 y	Melanoma ^§^	Questionnaire, death records, and linkage with cancer registries	Questionnaire and medical records	Never-users Ever-users	Ref. HR 1.46 (0.63–3.39)		Study site and calendar year of first infertility evaluation	7
Vessey and Yeates 2013 UK	Cohort Oxford/FPA *n* = 17,032 Age: 25–39 y Incident cases: 117 (in situ and invasive melanoma *) Follow-up: 628,000 woman-years	CMM: code 172 (ICD-8)	Hospital summaries	Interview	Never-users Ever-users Duration of use <4 years 5–6 years 7–8 years >8 years Time since last OC use Never-users ≤48 months 49–144 months 145–240 months 241–336 months ≥337 months	Ref. RR 0.8 (0.6–1.2) RR 0.6 (0.3–1.2) RR 0.7 (0.3–1.4) RR 1.0 (0.5–1.9) RR 1.0 (0.6–1.6) Ref. RR 0.4 (0.1–0.9) RR 1.4 (0.8–2.5) RR 0.7 (0.4–1.4) RR 0.9 (0.5–1.6) RR 0.7 (0.3–1.4)		Age, social class, smoking, and body mass index	7
Behrens et al. 2010 Multicentric (nine European countries)	Case–control Cases: 128 women diagnosed with uveal melanoma Age: 35–69 y Control: 1077 women	Uveal Melanoma	GP records	Interview	Never-users Ever-users	Ref. OR 0.94 (0.61–1.46)		Country, age group, frequency of lifetime ocular damage due to intense UV exposure, and eye color	5
Koomen et al. 2009 The Netherlands	Case–control Cases: 778 women diagnosed with CMM (invasive melanoma) Mean age: 53.6 y Control: 4072 Caucasian women Mean age: 54.6 y Age: >18y	CMM	Linkage with PALGA, the Dutch nationwide registry of histo- and cytopathology	PHARMO database	Never-users Ever-users (>0.5 year) Duration of use 1–700 days 701–1100 days >1100 days	Ref. OR 1.28 (1.06–1.54) OR 1.31 (0.96–1.77) OR 1.02 (0.75–1.40) OR 1.56 (1.16–2.10)	0.01 ≤0.01.	Total number of unique prescriptions dispensed (excluding estrogens) and use of nonsteroidal anti-inflammatory drugs	8
Lea et al. 2007 USA	Case–control Cases: 318 Caucasian women diagnosed with CMM (invasive melanoma) Control: 395 Caucasian women Age: 20–79 y	CMM	Histologic review	Interview	Never-users Ever-users	Ref. OR 1.3 (0.80–2.0)		Age group, education, study location, dysplastic nevus status, total number of nevi, extent of freckling, and never or ever pregnant	7
Hannaford et al. 2007 UK	Cohort Main dataset *n* = 49,950 women 83 incident cases (in situ and invasive melanoma *) RCGP *n* = 45,950 women 83 incident cases (in situ and invasive melanoma *)	CMM: code 172 (ICD-8)	National Health Service central registries in Scotland and England	GP records	Main dataset Never-users Ever-users RCGP Never-users Ever-users Duration of use ≤4 years 5–8 years >8 years Time since last OC use Current or <60 months 61–120 months 121–180 months 181–240 months ≥241 months	Ref. RR 0.92 (0.65–1.29) Ref. RR 1.03 (0.66–1.60) RR 0.95 (0.54–1.64 RR 0.79 (0.41–1.53) RR 1.71 (0.96–3.06) RR 1.17 (0.67–2.03) RR 1.57 (0.81–3.03) RR 0.51 (0.20–1.30) RR 0.89 (0.40–2.01) RR 0.62 (0.24–1.59)		Age, parity, smoking, social status, and ever use of hormone replacement therapy	8
Vessey and Painter 2006 UK	Cohort Oxford/FPA N= 17,032 Caucasian women Age: 25–39 y 94 incident cases (in situ and invasive melanoma *) Follow-up: until age 45 years	CMM: code 172 (ICD-8)	Hospital summaries	Interview	Never-users Ever-users Duration of use ≤4 years 5–8 years >8 years Time since last OC use Never-users ≤48 months 49–144 months 145–240 months ≥241 months	Ref. RR 0.8 (0.5–1.2) RR 0.4 (0.2–0.9) RR 0.9 (0.5–1.5) RR 1.0 (0.6–1.7) Ref. RR 0.4 (0.1–0.9) RR 1.4 (0.8–2.4) RR 0.6 (0.3–1.1) RR 0.8 (0.4–1.5)	n.s.	Age, social class, smoking, body mass index, parity, height, age at first-term pregnancy, and age at first marriage	7
Naldi et al. 2005 Italy	Case–control Cases: 316 women diagnosed with CMM (in situ and invasive melanoma *) Control: 308 women	CMM: codes 8720–8780 (ICD-O M)	Histological confirmation	Interview using a standard questionnaire	Never-users Ever-users	Ref. OR 1.05 (0.63–1.73)		Age, education, body mass index, number of melanocytic nevi, pigmentary traits, history of sunburns, and reaction to sun exposure	7
Freedman et al. 2003 USA	Cohort USRT N= 54,045 Caucasian women 159 incident cases (invasive melanoma) Follow-up: 698,028 person-years	CMM	Pathology reports and other confirmatory medical records	Mailed questionnaire	Never-users Ever-users Duration of use <5 years ≥5 years Status of OC use Never-users Past Current Age at first use <20 years 20–24 years ≥25 years Never-users	Ref. RR 1.2 (0.8–1.8) RR 1.8 (0.8–4.4) RR 1.2 (0.6–2.4) Ref. RR 1.2 (0.7–1.8) RR 1.4 (0.7–2.6) Ref. RR 1.0 (0.6–1.5) RR 1.1 (0.6–2.0) RR 0.9 (0.5–1.5)		Alcohol intake, years smoked, skin pigmentation, hair color, personal history of non-melanoma skin cancer, decade began work as a technologist, education, and proxy measures for residential childhood and adult sunlight exposure	5
Young et al. 2001 Australia	Case–control Cases: 14 women diagnosed with melanoma (in situ and invasive melanoma) Control: 85 women Age: 15-75 y	CMM	Queensland Cancer Registry and two other state cancer registries (New South Wales and Victoria)	Clinical records	Never-users Ever-users Duration of use <1 year 1–4 years 5–9 years ≥10 years	Ref. OR 0.94 (0.11–8.40) OR 1.78 (0.13-24.02) OR 1.74 (0.18-16.76) OR 0.22 (0.01–3.95) OR 0.53 (0.03–10.32)		Cohort entry year and entry age	7
Vessey et al. 2000 UK	Cohort Oxford/FPA *n* = 17,032 Caucasian women Age: 25–39 y 48 incident cases (in situ and invasive melanoma *) Follow-up: until age 45 years	CMM: code 172 (ICD-8)	Hospital referral	Interview	Never-users Ever-users Status of OC use Never-users Past Recently	Ref. RR 0.8 (0.4–1.4) Ref. RR 1.1 (0.6–2.0) RR 0.1 (0.0-0.6)		Age	6
Feskanich et al. 1999 USA	Cohort Nurses’ Health Study (NHS) *n* = 79,571 Caucasian premenopausal women Age: 30–55y 146 incident cases (invasive melanoma) Nurses’ Health Study II (NHS II) *n* = 104 122 Caucasian premenopausal women Age: 25–42 y 106 incident cases (invasive melanoma)	CMM	Medical records	Questionnaire	NHS + NHS II Never-users Ever-users Duration of use <5 years 5–9 years ≥10 years Time since last OC use <5 years 5–9.9 years 10–14.9 years ≥15 years Time since first OC use <10 years 10–19 years ≥20 years Age at first use <20 years 20–24 years ≥25 years Status of OC use Never-users Past Current NHS Never-users Ever-users Duration of use <5 years 5–9 years ≥10 years Status of OC use Never-users Past Current NHS II Never-users Ever-users Duration of use <5 years 5–9 years ≥10 years Status of OC use Never-users Past Current	Ref. RR 1.1 (0.8–1.5) RR 1.0 (0.7–1.4) RR 1.2 (0.8–1.9) RR 1.4 (0.8–2.5) RR 1.2 (0.7–2.0) RR 0.8 (0.5–1.3) RR 1.0 (0.7–1.6) RR 1.5 (0.9–2.5) RR 0.7 (0.4–1.4) RR 1.1 (0.8–1.5) RR 1.2 (0.7–1.9) RR 1.2 (0.7–2.2) RR 1.2 (0.8–1.8) RR 1.0 (0.7–1.4) Ref. RR 1.1 (0.8–1.5) RR 2.0 (1.2–3.4) Ref. RR 1.1 (0.7–1.5) RR 1.0 (0.7–1.5) RR 1.2 (0.7–2.1) RR 1.2 (0.6–2.7) Ref. RR 1.1 (0.7–1.5) RR 2.6 (1.2–5.6) Ref. RR 1.1 (0.6–2.0) RR 0.9 (0.5–1.7) RR 1.3 (0.6–2.5) RR 1.7 (0.8–3.7) Ref. RR 1.1 (0.6–2.0) RR 1.6 (0.8–3.3)	≤0.05 ≤0.05	Age, follow-up cycle, skin reaction after 2 h of sun exposure during childhood, number of sunburns over lifetime (NHS) or during teenage years (NHS II), number of moles on left arm (NHS) or on lower legs (NHS II), hair color, family history of melanoma, parity, height, and body mass index	5
Smith et al. 1998 USA	Case–control Cases: 308 Caucasian women diagnosed with melanoma (invasive melanoma) Control: 223 women Age: 15–75 y	CMM	Pathology reports and hospital tumor registry logs	Nurse interview	Never-users Ever-users Duration of use ≤2 years 2–5 years >5 years Age at first use ≤20 years 21–25 years ≥26 years	Ref. OR 1.10 (0.67–1.81) OR 1.26 (0.69–2.30) OR 0.60 (0.29–1.21) OR 1.44 (0.74–2.80) Ref. OR 1.39 (0.64–3.01) OR 0.95 (0.37–2.40)	0.956	Age, marital status, hair color, number of arm nevi, and sun exposure	8
Persson et al. 1996 Sweden	Cohort *n* = 22,579 women Mean age: 54.5 y 60 incident cases (in situ and invasive melanoma *) Follow-up: 15.5 y	CMM: code 190 (ICD-7) and code 172 (ICD-8)	National registration number linkage to the Central Cancer Registry	Pharmacy records	Never-users Ever-users	Ref. SIR 0.9 (0.7–1.1)		Age	8
Westerdahl et al. 1996 Sweden	Case–control Cases: 173 women diagnosed with malignant melanoma (invasive melanoma) Control: 280 women Age: 15–75 y	CMM	Regional Tumor Registry	Mailed questionnaire	Never-users Ever-users Duration of use <4 years 4–8 years >8 years	Ref. OR 2.2 (0.9–4.6) OR 1.5 (0.7–3.5) OR 1.0 (0.5–2.0)	0.7	Hair color (red, blond/fair, other), number of raised nevi (none, 1–3, >3), and number of sunburns (none, 1–2, >3)	6
Holly et al. 1995 USA	Case–control Cases: 452 Caucasian women with CMM (in situ and invasive melanom *) Controls: 930 Caucasian women Age: 25–59 y	CMM, SSM, and NM	Surveillance, Epidemiology, and End Results program	Interview	CMM Never-users Ever-users Duration of use <5 years 5–9 years ≥10 years Time since last OC use <5 years 5-9 years ≥10 years Time since first OC use ≤12 years 13–16 years ≥17 years SSM Never-users Ever-users Duration of use <5 years 5–9 years ≥10 years Time since last OC use <5 years 5–9 years ≥10 years Time since first OC use ≤12 years 13–16 years ≥17 years NM Never-users Ever-users Duration of use <5 years 5–9 years ≥10 years Time since last OC use <5 years 5–9 years ≥10 years Time since first OC use ≤12 years 13–16 years ≥17 years	Ref. OR 0.56 (0.42–0.76) OR 0.79 (0.56–1.1) OR 0.83 (0.54–1.3) OR 0.83 (0.55–1.2) OR 0.99 (0.68–1.4) OR 0.56 (0.42–0.75) OR 1.0 (0.70-1.6) OR 0.82 (0.57–1.2) OR 0.55 (0.41–0.74) Ref. OR 0.61 (0.43–0.85) OR 0.93 (0.64–1.4) OR 1.0 (0.64–1.6) OR 1.0 (0.67–1.6) OR 1.2 (0.78–1.8) OR 0.61 (0.44–0.85) OR 1.3 (0.84–2.0) OR 0.94 (0.63–1.4) OR 0.60 (0.43–0.84) Ref. OR 0.60 (0.31–1.1) OR 0.73 (0.34–1.6) OR 0.37 (0.11–1.3) OR 0.44 (0.15–1.2) OR 0.55 (0.22–1 4) OR 0.63 (0.34–1.2) OR 0.40 (0.14–1.2) OR 0.80 (0.36–1.8) OR 0.57 (0.30–1.1)		Age	6
Holly et al. 1994 USA	Case–control Cases: 452 Caucasian women with CMM (in situ and invasive melanoma *) Controls: 930 Caucasian women Age: 25–59 y	CMM, SSM, and NM	Surveillance, Epidemiology, and End Results program	Interview	CMM Never-users Ever-users Duration of use ≤0.5 years >0.5 years SSM Never-users Ever-users Duration of use ≤0.5 years >0.5 years NM Never-users Ever-users Duration of use ≤0.5 years >0.5 years	Ref. OR 1.0 (0.62–1.7) OR 0.81 (0.40–1.7) OR 1.3 (0.65–2.6) Ref. OR 1.2 (0.68–2.0) OR 0.83 (0.39–1.8) OR 1.6 (0.80–3.4) Ref. OR 0.64 (0.14-2.9) OR 0.64 (0.08–4.9) OR 0.65 (0.08–5.4)		Age and education	7
Zaridze et al. 1992 Russia	Case–control Cases: 96 women with CMM (in situ and invasive melanoma *) Controls: 96 women	CMM	All-Union Cancer Research Centre	Interview	Never-users Ever-users	Ref. OR 0.04 (0.003–0.53)	0.01	Skin color, freckles on arms, raised nevi on arms, nevi on trunk diameter >6 mm, and sunbathing at age 18–20	5
Lê et al. 1992 France	Case–control Cases: 57 Caucasian women with CMM (in situ and invasive melanoma *) Age: <45 y Controls: 65 Caucasian women	CMM	Histologically proven malignant melanoma	Interviewed during a period of hospitalization	Never-users Ever-users Duration of use 1–9 years ≥10 years Time since first OC use 1–14 years 15–20 years Age at first use <24 years ≥25 years	Ref. OR 1.0 (0.3–3.6) OR 2.4 (0.4–14.0) OR 0.9 (0.2–3.5) OR 2.0 (0.4–9.7) OR 1.2 (0.3–4.7) OR 1.2 (0.3–5.0)		Age at menarche, color of eyes, skin complexion, types of skin, and duration of sunlight exposure	4
Palmer et al. 1992 USA	Case–control Cases: 357 Caucasian women with CMM (severe invasive melanoma) Age: 18–64 y Controls: 2107 Caucasian women Age: <70 y	CMM, SSM, NM	Hospital records	Structured nurse interviews	SSM Never-users Ever-users Duration of use <1 year 1–4 years ≥5 years Time since last OC use <1 year 1–2 years 3–4 years 5–9 years ≥10 years Unknown Time since first OC use <1 year 1–4 years 5–9 years 10–14 years 15–19 years ≥20 years Unknown Status of OC use Current Age at first use <18 years 18–19 years 20–24 years ≥25 years Unknown NM Never-users Ever-users Duration of use <1 year 1–4 years ≥5 years Unknown/other types Never-users Ever-users Duration of use <1 year 1–4 years ≥5 years	Ref. RR 2.4 (1.2–4.6) RR 2.0 (1.1–3.6) RR 1.3 (0.6–2.6) RR 0.9 (0.4–2.2) RR 1.0 (0.5–2.0) RR 0.9 (0.4–1.8) RR 0.9 (0.6–1.4) RR 1.3 (0.9–1.8) RR 1.2 (0.7–2.0) RR 1.8 (0.7–4.6) RR 1.2 (0.6–2.4) RR 0.7 (0.4–1.2) RR 0.9 (0.6–1.5) RR 1.5 (1.0–2.2) RR 1.1 (0.7–1.8) RR 1.2 (0.6–2.4) RR 1.1 (0.6–2.1) RR 0.9 (0.4–1.8) RR 1.2 (0.7–2.1) RR 1.3 (0.9–1.9) RR 1.0 (0.7–1.4) RR 1.2 (0.6–2.4) Ref. RR 2.0 (0.8–4.8) RR 1.0 (0.4–2.5) RR 1.0 (0.4–2.6) Ref. RR 1.0 (0.6–1.7) RR 0.7 (0.5–1.2) RR 0.9 (0.5–1.5)		Age, geographic region, year of interview, years of education, religion, body mass index (kg/m), menopausal status, and skin type	6
Holly et al. 1991 USA	Case–control Cases: 186 Caucasian women with uveal melanoma Controls: 423 Caucasian women Age: 20–74 y	Uveal melanoma	Ocular Oncology Unit of the University of California	Telephone interview using a standard questionnaire	Never-users Ever-users Duration of use 1–3 years 4–9 years ≥10 years	Ref. RR 0.76 (0.48–1.20) RR 0.68 (0.37–1.24) RR 0.94 (0.53–1.64) RR 0.59 (0.28–1.27)		Age	7
Hannaford et al. 1991 UK	Cohort RCGP *n* = 23,000 women using OC + 23,000 never-users 58 incident cases (in situ and invasive melanoma *) Oxford/FPA *n* = 17,032 Caucasian women Age: 25–39 y 32 incident cases (in situ and invasive melanoma *)	CMM: code 172 (ICD-8)	RCGP: general practitioners’ records Oxford/FPA: hospital discharge records	RCGP: general practitioners’ records Oxford/FPA: post, telephone, or home visit	RCGP Never-users Ever-users Duration of use 1–4 years 5–9 years ≥10 years Oxford/FPA Never-users Ever-users Duration of use 1–4 years 5–9 years ≥10 years	Ref. RR 0.92 (0.55–1.54) RR 0.77 (0.41–1.45) RR 0.69 (0.31–1.52) RR 1.77 (0.80–3.90) Ref. RR 0.82 (0.38–1.76) RR 0.56 (0.16–1.63) RR 1.02 (0.37–2.56) RR 0.98 (0.24–3.09)		Age and parity at diagnosis, social class, and smoking habits at recruitment	8
Zanetti et al. 1990 Italy	Case–control Cases: 110 women with CM (in situ and invasive melanoma *) Controls: 123 women Age: 19–60 y	CMM, SSM	Turin Cancer Registry	Interview using a standard questionnaire	CMM Never-users Ever-users Duration of use <3 years ≥3 years SSM Never-users Ever-users	Ref. OR 0.98 (0.49–1.94) OR 0.94 (0.43–2.36) OR 0.98 (0.52–2.68) Ref. OR 1.26 (0.36–4.46)	0.58	Age, education, skin reaction to sun exposure, sunburns in childhood, and weeks of holiday on beach	6
Hartge et al. 1989 USA	Case–control Cases: 235 Caucasian women with intraocular malignant melanoma Mean age: 58.2 ± 15 y Controls: 220 women Mean age: 59.3 ± 14.4 y	Intraocular malignant melanoma	Ocular Oncology Service, with histopathological confirmation	Telephone interview	Never-users Ever-users Duration of use ≤1 year 2–9 years ≥10 years	Ref. RR 0.9 (0.4–1.7) RR 0.9 (0.3–2.4) RR 1.4 (0.5–4.3) RR 0.2 (0.3–1.2)	0.165	Age	5
Østerlind et al. 1988 Denmark	Case–control Cases: 278 women with CMM (not LMM) (in situ and invasive melanoma *) Controls: 536 women Age: 20–79 y	CMM (not LMM)	Danish Cancer Registry	Interview using a structured questionnaire	Never-users Ever-users Duration of use <2 years 2–4 years 5–9 years ≥10 years	Ref. RR 0.8 (0.6–1.1) RR 0.8 (0.4–1.4) RR 0.8 (0.4–1.3) RR 0.8 (0.4–1.4) RR 1.0 (0.6–1.7)	0.6	Age at diagnosis, host factors (naevi, freckles, and hair color), and sunbathing	7
Gallagher et al. 1986 Canada	Western Canada melanoma study Case–control Cases: 333 women with CMM (not lentigo maligna) (in situ and invasive melanoma *) Controls: 333 age-matched women Age: 20–69 y	CMM (not lentigo maligna), SSM, NM	Pathological slides and pathology reports	Interview using a standardized questionnaire	CMM Never-users Ever-users Duration of use <1 year 1–4 years ≥5 years SSM Never-users Ever-users Duration of use <1 year 1–4 years ≥5 years NM Never-users Ever-users Duration of use <1 year 1–4 years ≥5 years	Ref. RR 1.0 RR 0.9 RR 0.8 Ref. RR 1.1 RR 1.1 RR 0.9 Ref. RR 1.5 RR 1.0 RR 0.3		Skin color, hair color, freckling, and educational status	5
Gallagher et al. 1985 Canada	Western Canada melanoma study Case–control Cases: 333 women with CMM (not lentigo maligna) (in situ and invasive melanoma *) Controls: 333 age-matched women Age: 20–69 y	CMM (not lentigo maligna), SSM, NM,	Cancer registry	Interview	CMM Never-users Ever-users Duration of use <1 year 1–4 years ≥5 years SSM Never-users Ever-users Duration of use <1 year 1–4 years ≥5 years NM Never-users Ever-users Duration of use <1 year 1–4 years ≥5 years	Ref. RR 1.0 RR 0.9 RR 0.8 Ref. RR 1.1 RR 1.1 RR 0.9 Ref. RR 1.5 RR 1.0 RR 0.3	n.s. n.s. n.s.	Skin color, hair color, freckling, and educational status	5
Green and Bain 1985 Australia	Case–control Cases: 91 women with CMM (not LMM) (in situ and invasive melanoma) Controls: 91 age-matched women Age: 15–81 y	CMM (not LMM)	Histological report	Interview	Never-users Ever-users Time since last OC use ≤5 years 6–9 years ≥10 years Never-users Duration of use ≤ 4 years > 4 years	Ref. RR 0.7 (0.4–1.5) RR 0.5 (0.2–1.4) RR 0.8 (0.3–2.2) RR 0.9 (0.4–2.2) Ref. RR 1.0 (0.03–3.4) RR 0.4 (0.1–2.0)		Age, pigment phenotype, and exposure to sunlight	6
Holman et al. 1984 Australia	Case–control Cases: 276 women with CMM (in situ and invasive melanoma) Controls: 276 women Age: <80 y	CMM, HMF, SSM, UCM, NM	Histological report	Nurse interview administering the questionnaire “Environmental Lifestyle and Health”	CMM Never-users Ever-users Duration of use <2 years 2–4 years ≥5 years HMF Never-users Ever-users Duration of use <2 years ≥2 years SSM Never-users Ever-users Duration of use <2 years 2–4 years ≥5 years UCM Never-users Ever-users Duration of use <2 years 2–4 years ≥5 years NM Never-users Ever-users	Ref. OR 0.97 (0.59–1.61) OR 0.66 (0.37–1.19) OR 1.21 (0.65–2.23) OR 1.13 (0.62–2.04) Ref. OR 0.28 (0.03–2.60) OR 4.65 (0.54–40.40) Ref. OR 1.11 (0.56–2.19) OR 0.81 (0.39–1.67) OR 1.69 (0.73–3.93) OR 1.47 (0.67–3.20) Ref. OR 0.55 (0.14–2.25) OR 0.67 (0.20–2.28) OR 0.75 (0.20–2.81) Ref. OR 0.33 (0.02–3.56)	0.903 0.251 0.145 0.871 0.177 0.802 0.617	None	5
Helmrich et al. 1984 USA	Case–control Cases: 160 women with CMM (in situ and invasive melanoma) Median age: 42 y Controls: 640 age-matched women Median age: 42 y Age: 20–59 y	CMM	Hospital discharge and pathology records	Nurse interview administering a standard questionnaire	Never-users Ever-users Duration of use <1 year 1–4 years 5–9 years ≥10 years	Ref. RR 0.8 (0.5–1.3) RR 0.7 (0.4–1.3) RR 0.8 (0.5–1.4) RR 0.8 (0.4–1.7) RR 1.0 (0.4–2.9)		Age, geographic area, religion, years of education, and date of interview	6
Beral et al. 1984 Australia	Case–control Cases: 287 Caucasian women attending the melanoma clinic at Sidney Hospital (in situ and invasive melanoma *) Controls: 574 age-matched women Age: 18–54 y	CMM	Classification of biopsy and histological features	Interview using a standard questionnaire	Never-users Ever-users Duration of use ≥5 years	Ref. RR 1.0 RR 1.5	<0.05	None	3
Holly et al. 1983 USA	Case–control Cases: 42 SSM, 68 CMM (in situ and invasive melanoma *) in Caucasian women Controls: 592 Caucasian women Age: 37–60 y	CMM and SSM	Cancer Surveillance System	Interview	CMM Never-users Ever-users Duration of use 1–4 years 5–9 years ≥10 years SSM Never-users Ever-users Duration of use 1–4 years 5–9 years ≥10 years	Ref. RR 1.0 RR 1.5 RR 2.1 Ref. RR 0.73 RR 2.4 RR 3.6	0.09 0.004	Age Age at birth of first child	5
Bain et al. 1982 USA	Case–control Cases: 141 nurses (in situ and invasive melanoma *) Controls: 2820 nurses Age: 30–55 y	CMM	Pathology report or hospital discharge notes.	Postal questionnaire	Never-users Ever-users Duration of use 1–24 months ≥25 months	Ref. RR 0.82 (0.53–1.27) RR 0.84 (0.47–1.49) RR 0.83 (0.47–1.46)		Age at diagnosis, state of residence, parity, age at first pregnancy, height, and prior hair dye use	5
Adam et al. 1981 UK	Case–control Cases: 169 Caucasian women (in situ and invasive melanoma *) Controls: 507 Caucasian women Age: 15–49 y	CMM	Cancer registries in the Oxford Regions and the South Western Region; cases verified with hospital notes and GP records	Postal questionnaire GP records	Postal questionnaire data: Never-users Ever-users Duration of use ≥5 years GP records Never-users Ever-users	Ref. RR 1.13 (0.73–1.75) RR 1.59 (0.83–3.03) Ref. RR 1.34 (0.92–1.96)		None	5
Kay 1981 UK	Prospective study (in situ and invasive melanoma *)	CMM: code 172 (ICD)	–	–	Never-users Ever-users	Ref. RR 1.46 (0.73–2.91)		None	0

Abbreviations: ALM—acro-lentiginous melanoma; CMM—cutaneous malignant melanoma; EPIC—European Prospective Investigation into Cancer and Nutrition; GP—general practitioner; HMF—Hutchinson’s melanotic freckle; HR—hazard ratio; ICD-O-2—International Classification of Diseases for Oncology, Second Edition; ICD-O-3—International Classification of Diseases for Oncology, Third Edition; ICD-O-M—International Classification of Diseases for Oncology Morphology; ICD-7—International Classification of Diseases, 7th revision; ICD-8—International Classification of Diseases, 8th revision; LMM—lentigo maligna melanoma; NOS—Newcastle–Ottawa scale; NHS—Nurses’ Health Study; NHS II—Nurses’ Health Study II; NIH-AARP—National Institutes of Health American Association of Retired Persons Diet and Health Study; NM—nodular melanoma; n.s.—not significant; OCs—oral contraceptives; OR—odds ratio; Oxford FPA—Oxford Family Planning Association; RCGP—Royal College of General Practitioners; RR—relative risk; SSM—superficial spreading melanoma; UCM—unclassifiable cutaneous melanoma; USRT—United States Radiologic Technologist; UV—ultraviolet. * We assumed the inclusion of both in situ and invasive melanoma since the morphology behavior is not specified in the text. ^§^ Cutaneous not specified.

**Table A2:** Main characteristics of studies included in the systematic review and meta-analysis on menopausal hormone therapy (MHT) and malignant melanoma risk.

First Author Year Location	Study Design Name and Population Cases/Controls Follow-Up Incident Cases Age	Tumor Characteristics	Identification of Cases	Exposure Assessment	MHT Exposure Duration of Use Status of MHT Use Regimen Type of MHT Route of Administration	OR/RR/HR/SIR (95% CI)	*p* for Trend	Matched or Adjusted Variables	NOS Score
Stoer et al. 2021 Norway	Cohort study NOWAC 70733 Age: 30–75 y 392 (incidence 0.019) Follow-up: 16 y	CMM	Linkage with Cancer Registry of Norway	Questionnaire	Never use Ever ET EPT Current ET EPT Past	Ref HR 0.97 (0.63–1.49) HR 1.04 (0.87–1.24) HR 1.36 (0.96–1.94) HR 1.11 (0.91–1.36) HR 0.90 (0.72–1.11)		Age, marital status, hair color, skin color, large asymmetric caevi on the legs, age at menarche, menstrual cycle length, parity, oral contraceptive use, sunburns, bathing vacations, and solarium use	8
Olsen et al. 2021 Australia	Cohort study Qskin Sun and Health Study 21068 Age: 40–69 y 392 (incidence 0.019) Follow-up: 5.4 y	invasive CMM and all CMM (invasive and in situ)	Linkage with Quennesland Cancer Registry	Survey Qskin Sun and Helath	MHT use No Yes Duration of MHT use 0–6 months 7–26 months 27–60 >60	Ref 1.13 (0.78–1.66) Ref HR 0.90 (0.37–1.63) HR 1.67 (0.99–2.71) HR 0.95 (0.57–12.58)	0.8	Age, highest level of education achieved, body mass index, smoking status, and measure of health service use	8
Cervenka et al. 2020 10 European country	Cohort study EPIC *n* = 134,758 postmenopausal women Age: 51.1 ± 9.7 years 770 incident cases (in situ and invasive melanoma) Follow-up: 13.9 years	CMM: C44 (ICD-O-2)	Linkage with population cancer and pathology registries, health insurance and hospital discharge records, national and regional mortality registries, and active follow-up through contacts with participants and their next of kin	Country-specific questionnaire	Never-users Ever-users Duration of use ≤5 years >5 years Status of MHT use Current Past Unknown Regimen Continuous Type of MHT ET EPT Other/Unknown Route of administration Oral Cutaneous Other/Unknown Cutaneous: Cream Patch	Ref. HR 1.14 (0.97–1.35) HR 1.12 (0.93–1.34) HR 1.05 (0.80–1.36) HR 1.18 (0.98–1.43) HR 1.07 (0.86–1.34) HR 1.36 (0.72–2.59) HR 0.88 (0.55–1.41) HR 1.24 (0.93–1.64) HR 1.18 (0.94–1.48) HR 1.04 (0.71–1.53) HR 1.46 (0.99–2.16) HR 1.25 (0.76–2.04) HR 0.91 (0.52–1.59) HR 2.20 (1.12–4.29) HR 0.84 (0.41–1.70)	0.42	Center, age at recruitment, education, age at menarche, length of menstrual cycles, number of full-term pregnancies, oral contraceptive use, height, body mass index, and tobacco use	7
Hicks et al. 2019 Denmark	Nested case–control Age: 45–85 years Cases: 8279 (invasive melanoma) Controls: 165,580	CMM	Danish Cancer Registry	Prescriptions in nationwide registry sources	Non-users (patients who did not use HRT (excluding intravaginal estrogens) in the 5 years prior to diagnosis and to 1 year after diagnosis) Ever-users Route of administration Oral Transdermal	Ref. OR 1.18 (1.12–1.24) OR 1.18 (1.10–1.26) OR 1.37 (1.17–1.61)		Age, calendar time, drugs suggested to have photosensitizing properties, oral contraceptive, low-dose aspirin, non-steroidal anti-inflammatory drugs, statins, diabetes, chronic obstructive pulmonary disease, chronic renal insufficiency, diseases associated with heavy alcohol consumption, inflammatory bowel disease, psoriasis, sarcoidosis and stroke, modified Charlson Comorbidity Index, and highest achieved education	9
Botteri et al. 2019 Finland	Cohort N= 293,570 Age: 59.2 years 1695 incident cases (invasive melanoma) Follow-up: 15.6 years	CMM: C44 (ICD-O-3 topography), 872–9 (ICD-O-3 morphology) and behavior 3	Finnish Cancer Registry	Medical Reimbursement Register of the National Social Insurance Institution	Duration of use ≥6 months ≥60 months	SIR 1.16 (1.11–1.22) SIR 1.28 (1.19–1.36)		Age	7
Cervenka et al. 2019 France	Cohort E3N (Etude Epidémiologique auprès de femmes de l’Education Nationale) *n* = 75,523 postmenopausal women 539 incident cases (in situ and invasive melanoma *) Median follow-up: 10.4 years	CMM	French National Cancer Institute	Questionnaire	Never-users Ever-users Duration of use <2.5 years 2.5–5.2 years 5.3–8.3 years ≥8.4 years Status of MHT use Recent Past Type of MHT ET EPT (estrogen combined with a progestogen or an androgen) Low-potency estrogens Other/Unknown Route of administration Oral Transcutaneous Other or multiple	Ref. HR 1.35 (1.07–1.71) Ref. HR 1.14 (0.83–1.56) HR 1.07 (0.75–1.53) HR 1.11 (0.77–1.61) HR 1.26 (0.98–1.61) HR 1.55 (1.17–2.07) HR 1.49 (0.95–2.34) HR 1.36 (1.05–1.77) HR 0.79 (0.41–1.53) HR 1.90 (1.30–2.78) HR 1.32 (0.86–2.01) HR 1.35 (1.06–1.74) HR 1.36 (1.02–1.81)	0.43	Age, stratified according to year of birth and adjusted for residential UV exposure at birth and at inclusion, pigmentary traits, and family history of skin cancer	7
		SSM, NM, LMM, ALM, other			SSM: Never-users Ever-users NM: Never-users Ever-users LMM: Never-users Ever-users ALM: Never-users Ever-users Other types: Never-users Ever-users	Ref. HR 1.42 (1.04–1.93) Ref. HR 0.56 (0.15–2.11) Ref. HR 2.57 (1.16–5.86) Ref. HR 1.91 (0.59–6.16) Ref. HR 0.96 (0.56–1.66)		Residential UV exposure, pigmentary traits, and family history of skin cancer	
Donley et al. 2019 USA	Cohort NIH-AARP *n*= 167,503 Postmenopausal women Age: 62.2 ± 5.3 years 1061 incident cases (invasive melanoma) Follow-up: 15.5 years	CMM: C44.0–C44.9 (ICD-O-3 Topography) and 8720-8780 (ICD-O-3 M)	Record linkage with state cancer registries	Baseline questionnaire	Never-users Ever-users Duration of use 1–4 years 5–9 years ≥10 years	Ref. HR 1.02 (0.89–1.15) HR 0.95 (0.80–1.13) HR 1.10 (0.92–1.33) HR 1.01 (0.86–1.18)	0.68	Age, ambient ultraviolet radiation, education, body mass index, smoking status, marriage, family history of cancer, colonoscopy, or sigmoidoscopy	7
Botteri et al. 2017 Norway	Cohort *n* = 694,696 women 1476 incident cases (in situ and invasive melanoma *) Median follow-up: 4.8 years	CMM code 190 (ICD-7)	Cancer Registry of Norway	Norwegian Prescription Database	Never-users Ever-users Status of MHT use Current Past Regimen Continuous Type of MHT ET EPT Mixed users	Ref. RR 1.15 (1.00–1.32) RR 1.19 (1.03–1.37) RR 1.00 (0.82–1.21) RR 0.80 (0.59–1.09) RR 1.45 (1.21–1.73) RR 0.91 (0.70–1.19) RR 0.94 (0.68–1.31)		Age, number of children, age at first birth, education, marital status, sun exposure, use of antihypertensives, antidiabetics, statins, and thyroid therapy	7
Simin et al. 2017 Sweden	Cohort *n* = 290,186 MHT ever-users Age: ≥40 years 898 incident cases (in situ and invasive melanoma *)	CMM C43.0-9 (ICD-10)	Cancer Registry	Swedish Prescribed Drug Registry	Never-users Ever-users Type of MHT ET EPT	Ref. SIR 1.19 (1.11-1.27) SIR 1.26 (1.15-1.39) SIR 1.13 (1.02-1.24)	0.000	Age	6
Brinton et al. 2015 USA	Cohort *n* = 9892 women 70 incident cases Median follow-up: 30 years	Melanoma ^§^	Questionnaire, death records, and linkage with cancer registries	Questionnaire and medical records	Never-users Ever-users	Ref. HR 1.10 (0.63–1.94)		Study site and calendar year of first infertility evaluation	7
Behrens et al. 2010 Multicentric	Case–control Cases: 77 women diagnosed with uveal melanoma Age: 55–69 years Control: 596 women	Uveal melanoma	GP records	Interview	Never-users Ever-users	Ref. OR 1.44 (0.74–2.80)		Country, age group, frequency of lifetime ocular damage due to intense UV exposure, and eye color	5
Koomen et al. 2009 The Netherlands	Case–control Cases: 778 women diagnosed with CMM (invasive melanoma) Mean age: 53.6 years Control: 4072 Caucasian women Mean age: 54.6 years Age: >18 years	CMM	Linkage with PALGA, the Dutch nationwide registry of histology and cytopathology	PHARMO database	Never-users Ever-users (>0.5 year) Duration of use 1–671 days > 671 days	Ref. OR 2.08 (1.37–3.14) OR 2.16 (1.24–3.78) OR 1.98 (1.08–3.62)		Total number of unique prescriptions dispensed (excluding estrogens) and use of nonsteroidal anti-inflammatory drugs	8
Lea et al. 2007 USA	Case–control Cases: 318 Caucasian women diagnosed with CMM (invasive melanoma) Control: 395 Caucasian women Age: 20–79 years	CMM	Histologic review	Interview	Never-users Ever-users	Ref. OR 0.80 (0.45–1.4)		Age group, education, study location, dysplastic nevus status, total number of nevi, extent of freckling, and never or ever pregnant	7
Naldi et al. 2005 Italy	Case–control Cases: 316 women diagnosed with CMM (in situ and invasive melanoma *) Control: 308 women	CMM 8720–8780 (ICD-O M)	Histological confirmation	Interview using a standard questionnaire	Never-users Ever-users	Ref. OR 1.05 (0.49–2.26)		Age, education, body mass index, number of melanocytic nevi, pigmentary traits, history of sunburns, and reaction to sun exposure	7
Freedman et al. 2003 USA	Cohort USRT *n* = 54,045 Caucasian women 159 incident cases (invasive melanoma)	CMM	Pathology reports and other confirmatory medical records	Mailed questionnaire	Never-users Ever-users	Ref. RR 1.2 (0.8–1.8)		Alcohol intake, years smoked, skin pigmentation, hair color, personal history of non-melanoma skin cancer, decade began work as a technologist, education, and proxy measures for residential childhood and adult sunlight exposure	5
Holly et al. 1994 USA	Case–control Cases: 452 Caucasian women with CMM (in situ and invasive melanoma *) Controls: 930 Caucasian women Age: 25–59 years	CMM, SSM, and NM	Surveillance, Epidemiology, and End Results program	Interview	CMM: Never-users Ever-users Duration of use ≤2 years >2 years SSM: Never-users Ever-users Duration of use ≤2 years >2 years NM: Never-users Ever-users Duration of use ≤2 years >2 years	Ref. OR 0.88 (0.50–1.6) OR 0.71 (0.34–1.5) OR 1.2 (0.56–2.4) Ref. OR 1.0 (0.54–2.0) OR 0.85 (0.38–1.9) OR 1.3 (0.58–3.0) Ref. OR 0.82 (0.26–2.6) OR 0.27 (0.03–2.2) OR 1.8 (0.49–6.8)		Age and education	7
Holly et al. 1991 USA	Case–control Cases: 142 Caucasian women with uveal melanoma Controls: 323 Caucasian women Age: 20–74 years	Uveal melanoma	Ocular Oncology Unit of the University of California	Phone interview using a standard questionnaire	Never-users Ever-users Duration of use ≤1 year 2–9 years ≥10 years	Ref. RR 0.85 (0.57–1.26) RR 0.71 (0.35–1.46) RR 0.68 (0.40–1.15) RR 1.14 (0.69–1.90)		Age	6
Adami et al. 1989 Sweden	Cohort *n* = 23,244 Postmenopausal women 31 incident cases (in situ and invasive melanoma *) Follow-up: 6.7 years	CMM: 190 (ICD-7)	National Cancer Registry	Questionnaire by mail	Never-users Ever-users Type of MHT ET Other	Ref. RR 1.45 (0.99–2.06) RR 1.3 (0.8–2.0) RR 1.9 (1.0–3.5)		None	4
Hartge et al. 1989 USA	Case–control Cases: 214 Caucasian women with intraocular malignant melanoma Mean age: 58.2 ± 15 years Controls: 209 women Mean age: 59.3 ± 14.4 years	Intraocular malignant melanoma	Ocular Oncology Service, with histopathological confirmation	Telephone interview	Never-users Ever-users Duration of use ≤1 year 2–5 years ≥6 years	Ref. RR 2.0 (1.2–3.1) RR 1.9 (1.0–3.5) RR 1.6 (0.7–3.8) RR 2.2 (0.9–5.8)	0.08	Age and history of oophorectomy	5
Østerlind et al.1988 Denmark	Case–control Cases: 209 women with CMM (not LMM) (in situ and invasive melanoma *) Controls: 411 women Age: 20–79 years	CMM (not LMM)	Danish Cancer Registry	Interview using a structured questionnaire	Never-users Ever-users Duration of use <2 years 2–6 years ≥7 years Type of MHT ET EPT	Ref. RR 1.1 (0.7–1.7) RR 0.8 (0.3–1.7) RR 1.2 (0.6–2.8) RR 1.2 (0.7–2.2) RR 1.3 (0.8–2.1) RR 1.5 (0.8–2.8)		Age at diagnosis, naevi, and sunbathing	7
Beral et al. 1984 Australia	Case–control Cases: 287 Caucasian women attending the melanoma clinic at Sidney Hospital (in situ and invasive melanoma *) Controls: 574 age-matched women Age: 18–54 years	CMM	Classification of biopsy and histological features	Interview using a standard questionnaire	Never-users Ever-users	Ref. RR 1.4 (0.78–2.61)		None	3
Holly et al. 1983 USA	Case–control Caucasian women Cases: CMM (in situ and invasive melanoma *) Age: 45–74 years	CMM and SSM	Cancer Surveillance System	Interview	CMM: Never-users Duration of use 1–3 years 4–7 years ≥8 years SSM: Never-users Duration of use 1–3 years 4–7 years ≥8 years	Ref. RR 1.1 RR 0.85 RR 1.0 Ref. RR 1.1 RR 1.1 RR 0.98	0.88 0.94	Age	5

Abbreviations: ALM—acro-lentiginous melanoma; CMM—cutaneous malignant melanoma; EPIC—European Prospective Investigation into Cancer and Nutrition; EPT—estrogen–progestin therapy; ET—estrogen therapy; GP—general practitioner; HR—hazard ratio; ICD-O-2—International Classification of Diseases for Oncology, Second Edition; ICD-O-3—International Classification of Diseases for Oncology, Third Edition; ICD-O M—International Classification of Diseases for Oncology Morphology; ICD-7—International Classification of Diseases, 7th Revision; ICD-10—International Classification of Diseases, 10th Revision; LMM—lentigo maligna melanoma; MHT—menopausal hormone therapy; NIH-AARP—National Institutes of Health American Association of Retired Persons Diet and Health Study; NM—nodular Melanoma; NOS—Newcastle–Ottawa scale; OR—odds ratio; Oxford FPA—Oxford Family Planning Association; RR—relative risk; SSM—superficial skin melanoma; SIR—standardized incidence rate; USRT—United States Radiologic Technologist; UV—ultraviolet. * We assumed the inclusion of both in situ and invasive melanoma since the morphology behavior is not specified in the text. ^§^ Cutaneous not specified.
